# Lifetime of racetrack skyrmions

**DOI:** 10.1038/s41598-018-21623-3

**Published:** 2018-02-21

**Authors:** Pavel F. Bessarab, Gideon P. Müller, Igor S. Lobanov, Filipp N. Rybakov, Nikolai S. Kiselev, Hannes Jónsson, Valery M. Uzdin, Stefan Blügel, Lars Bergqvist, Anna Delin

**Affiliations:** 1 Science Institute of the University of Iceland, 107 Reykjavik, Iceland; 2 ITMO University, 197101 St. Petersburg, Russia; 3 Peter Grünberg Institut and Institute for Advanced Simulation, Forschungszentrum Jülich and JARA, D-52425 Jülich, Germany; 4 Department of Physics, KTH Royal Institute of Technology, Stockholm, SE-10691 Sweden; 5 Aalto University, FI-00076 Espoo, Finland; 6 Department of Physics, St. Petersburg State University, St. Petersburg, 198504, Russia ITMO University, 197101 St. Petersburg, Russia; 7 Department of Applied Physics, School of Engineering Sciences, KTH Royal Institute of Technology, Electrum 229, SE-16440 Kista, Sweden; 8 SeRC (Swedish e-Science Research Center), KTH Royal Institute of Technology, SE-10044 Stockholm, Sweden; 9 Department of Physics and Astronomy, Uppsala University, Box 516, SE-75120 Uppsala, Sweden

## Abstract

The skyrmion racetrack is a promising concept for future information technology. There, binary bits are carried by nanoscale spin swirls–skyrmions–driven along magnetic strips. Stability of the skyrmions is a critical issue for realising this technology. Here we demonstrate that the racetrack skyrmion lifetime can be calculated from first principles as a function of temperature, magnetic field and track width. Our method combines harmonic transition state theory extended to include Goldstone modes, with an atomistic spin Hamiltonian parametrized from density functional theory calculations. We demonstrate that two annihilation mechanisms contribute to the skyrmion stability: At low external magnetic field, escape through the track boundary prevails, but a crossover field exists, above which the collapse in the interior becomes dominant. Considering a Pd/Fe bilayer on an Ir(111) substrate as a well-established model system, the calculated skyrmion lifetime is found to be consistent with reported experimental measurements. Our simulations also show that the Arrhenius pre-exponential factor of escape depends only weakly on the external magnetic field, whereas the pre-exponential factor for collapse is strongly field dependent. Our results open the door for predictive simulations, free from empirical parameters, to aid the design of skyrmion-based information technology.

## Introduction

Spin textures with topological charge, also called skyrmions^1–3^, hold great promise as a basis for a new type of information technology^4–6^. In particular, information flow can be associated with metastable skyrmions driven along a magnetic strip, as suggested in skyrmion racetrack schemes^5,6^. It has been demonstrated that skyrmions are sensitive to controlled external stimuli such as electric current^7–9^, which is beneficial for efficient, low power data processing. For such a technology to be viable, however, the skyrmion lifetime, *τ*, is an essential quantity. It is a quantitative measure of stability and needs to be long enough to enable information storage with negligible loss. Prediction of the lifetime of skyrmions in arbitrary materials and materials combinations as a function of temperature and various external parameters such as applied magnetic field is thus of central importance for developing an optimized skyrmion-based technology. Although skyrmions owe their stability to topology, the lifetime cannot be derived from topological considerations *per se*. The celebrated notion of topological protection of a single skyrmion localized in a ferromagnetic ground state of infinite spatial dimensions described in the language of continuum field theory with fixed magnetization length translates to energy barriers, whose heights become finite for physical systems and described in practice by the escape of the skyrmion to the ferromagnetic state by radial collapse, or through the system boundary.

**Figure 1:**
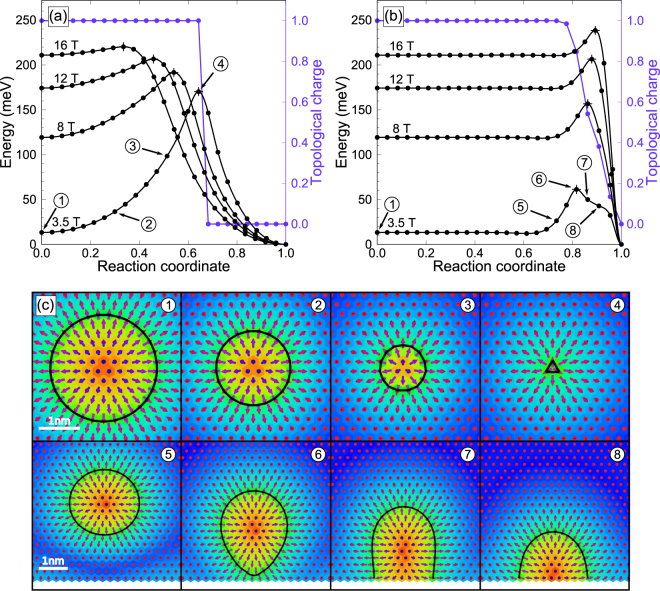
Mechanisms of the skyrmion annihilation in a fcc-Pd/Fe/Ir(111) racetrack. Energy variation along the MEPs for radial collapse of the skyrmion in the interior of the strip (**a**) and escape of the skyrmion through the boundary (**b**), shown for four values of applied magnetic field. The filled circles show position of the intermediate states along the annihilation paths, while crosses indicate energy maxima along the MEPs. Variation of the absolute value of the topological charge along the MEPs is represented by a purple line for *B* = 3.5 T. The reaction coordinate is defined as the normalized displacement along the MEP. The starting- and end-points of the reaction coordinate are the skyrmion and ferromagnetic states, respectively. The encircled numbers label the states for which spin configurations are shown in the lower panel (**c**). The background color indicates the value of the out-of-plane component of the magnetic vectors (red ↔ up, blue ↔ down). Black solid lines show the contour where the out-of-plane component of magnetization vanishes.

Here, we show that it is indeed possible to calculate–from first principles–the lifetime of skyrmions. To demonstrate our method, we present results for an fcc-stacked film of one monolayer of Pd and Fe on an Ir(111) substrate, one of the best investigated systems hosting single Néel-type skyrmions stabilized by interface generated Dzyaloshinskii-Moriya (DM) interaction^10,11^. We compare the results to an hcp-stacked Pd film on Fe/Ir(111), which emerges experimentally as a structurally metastable state^12^. The spin textures appearing in PdFe/Ir(111) system as a function of applied magnetic field have been characterized using spin-polarized scanning tunneling microscopy at low temperature^13^. At zero and low applied magnetic field, this system exhibits a spin-spiral state with a period of 6–7 nm, see Fig. 1, panels A and D in ref.^13^. As the magnetic field is increased to about 1 T, skyrmions start to form (Fig. 1, panel E in ref.^13^). The observed skyrmions are quite small, with a diameter of just a few nanometers. At even higher field, the spin-spirals disappear and a pure hexagonal skyrmion lattice emerges (Fig. 1, panel F in ref.^13^). Finally, at field above 2 T, a field-polarized ferromagnetic phase is observed with isolated skyrmions pinned at atomic defects (see Fig. 1, panel G in ref.^13^). This sequence of phases as a function of applied magnetic field can be reproduced by an atomistic spin Hamiltonian (see Methods section) parameterized from first principles density functional theory (DFT) calculations^14–17^. The calculations predict that the ferromagnetic phase emerges at 0.5 T (3.2 T) for hcp (fcc) stacking of the Pd layer, while the skyrmion size ranges from 2 to 3 nm at 4 T, depending on the Pd layer stacking^17^. These theoretical results are in good agreement with the experimental data^12,13^. The critical temperature of the phase transition to the paramagnetic state was calculated to be between 227 K^18^ and 250 K^16^, for independently determined microscopic parameter sets. Furthermore, these temperatures were found to be independent of the magnetic field strength^18^. However, the temperature range at which skyrmions in this system become stable on macroscopic time scales remains unexplored, although some rough estimates obtained by extrapolation of Monte Carlo and spin dynamics simulations to low temperatures exist^16,19^.

Our approach to the identification of skyrmion lifetimes combines an atomistic Hamiltonian parametrized from first principles and statistical rate theory, which provides a solution to the rare-event problem. The rare-event problem arises from the fact that in the relevant temperature range, transitions between stable magnetic states induced by thermal fluctuations, e.g. a skyrmion collapsing to the ferromagnetic phase, are typically rare events on the intrinsic time scale of the magnetization dynamics of the system and makes direct simulations of finite temperature spin dynamics^16,20^ an intractable approach to evaluate the lifetimes. On the other hand, it is this separation of time scales that makes it possible to apply statistical methods (see Methods section).

Rate theories^21–24^ predict an Arrhenius expression for the transition rate *k* as a function of temperature *T*,

1 $$k(T)=\frac{1}{\tau (T)}=\nu (T){e}^{-{\rm{\Delta }}E/{k}_{{\rm{B}}}T},$$

where the magnitudes of both the activation energy Δ*E* and the entropic pre-exponential factor also referred to as the attempt frequency *ν* depend on the parameters of the system as well as the mechanism of the magnetic transition.

The mechanism and energy barrier of skyrmion annihilation in magnetic monolayers have been identified previously^25,26^. It involves symmetrical shrinking and collapse of the skyrmion via an intermediate state reminiscent of the cross section texture of a Bloch point. In finite-size systems, the boundaries may provide additional paths for the skyrmion creation and annihilation. Quantitative assessment of the effect of boundaries is particularly important in the context of the racetrack memory device, where skyrmions need to be guided reliably along a magnetic strip^5^. While the repulsive interaction between skyrmions and edges has been pointed out before^27,28^, and energy barriers for the skyrmion escape through the system boundary have been evaluated^29–31^, the impact of the boundaries on the skyrmion lifetime at a finite temperature is lacking.

Most previous work on skyrmion stability has relied on an effective, nearest-neighbor approximation for the interatomic magnetic exchange interaction entering the atomistic spin Hamiltonian. This approach is sufficient for the description of equilibrium properties of skyrmions such as size and shape as well as zero-temperature phase diagrams. However, a recent study of skyrmion stability in the Pd/Fe/Ir(111) system^17^ employing *ab initio* calculations of shell-resolved exchange interaction parameters and identification of minimum energy paths for the skyrmion annihilation has demonstrated that a nearest-neighbor description of magnetic interactions greatly underestimates the energy barrier for skyrmion collapse. Therefore, including terms beyond nearest-neighbor pairwise interaction is critical for the quantitative description of the skyrmion stability in itinerant electron magnets, for which long-range, frustrated exchange is a typical feature.

The evaluation of the attempt frequency *ν* is essential for the calculation of the skyrmion lifetime. While the activation energy defines strong, exponential dependence of the lifetime on temperature, it is the attempt frequency that establishes the timescale. It can vary by several orders of magnitude depending on the parameters of the magnetic system, as has been demonstrated both experimentally and theoretically for Fe islands on W(110)^32,33^. Therefore, assuming *ν* to have some fixed value independent of the system annihilation mechanism will lead to incorrect results, since the entropic and dynamical contributions are then not accounted for correctly. Based on Monte-Carlo simulations, Hagemeister *et al*.^19^ concluded that the attempt frequency for skyrmion annihilation was orders of magnitude smaller than that for skyrmion creation due to a broader shape of the skyrmion state energy minimum. An unusually small magnitude of the attempt frequency for thermally-activated skyrmion annihilation, on the order of 10^9^–10^10^ s^−1^, was confirmed by Rózsa *et al*.^16^ and Rohart *et al*.^20^ who studied the skyrmion stability using Langevin spin dynamics simulations at relatively high temperatures.

In the present work, we predict the lifetime of skyrmions in the Pd/Fe/Ir(111) system by evaluating the pre-exponential factor *ν* as well as the activation energy Δ*E* using harmonic transition state theory for spins^24^ and an atomistic spin Hamiltonian parametrized from DFT calculations. Our approach does not rely on direct simulations of finite-temperature spin dynamics, and, therefore, does not suffer from the problem of rare skyrmion annihilation events, making it possible to quantitatively describe stability of long-lived skyrmions. We show that the pre-exponential factor acquires a temperature dependence due to the presence of Goldstone modes, i.e. degrees of freedom for which the energy is constant. We find that the lifetime of an isolated skyrmion in a magnetic strip is governed by at least two annihilation mechanisms: Escape through the boundary and radial collapse in the interior. We identify the crossover from one mechanism to the other as a function of applied magnetic field and temperature. While the calculations were performed for the Pd/Fe/Ir(111) system, the cross-over effect we predict should be a general feature for skyrmions in finite-size geometries and needs to be taken into account when designing novel logic or memory devices based on racetrack skyrmions.

## Results

### Skyrmion annihilation mechanisms in a racetrack

Figure 1 shows results of calculations of skyrmion annihilation in a Pd/Fe/Ir(111) racetrack geometry (see Methods section for the detailed description of the simulated system). Minimum energy paths (MEPs) between local energy minima corresponding to the skyrmion state and the field-polarized, ferromagnetic configuration are shown for different applied magnetic fields (see Methods section for the details of MEP calculations). The calculations were carried out for magnetic fields at which the field-polarized ferromagnetic configuration is the lower energy state, but individual skyrmions exist as metastable long-lived quasiparticles^17^. For fcc-Pd/Fe/Ir(111), metastable isolated skyrmions within the saturated state are realized above a critical field ${B}_{{\rm{F}}}^{{\rm{fcc}}}\approx 3.2$ T at which the skyrmion state and the ferromagnetic state are energetically degenerate (see Fig. 1 in ref.^17^ for the full phase diagram of the system as a function of applied magnetic field). This field value is consistent with measurements^12,34^, although exact phase boundaries have not yet been investigated experimentally. For hcp-Pd/Fe/Ir(111), the ferromagnetic phase is the ground state of the system over the whole range of magnetic field values, but skyrmions are metastable when the field is larger than ${B}_{{\rm{F}}}^{{\rm{hcp}}}\approx 0.5$ T (see ref.^17^). The MEP calculations revealed two transition mechanisms, which are the same for both stackings of the Pd layer. The mechanisms are shown in Fig. 1 for the fcc-Pd/Fe strip on Ir(111) (results for the hcp-Pd/Fe strip are presented in Supplementary Fig. S1). The first mechanism is characterized by a radial collapse of the skyrmion in the interior of the strip. It involves a symmetrical rotation of spins causing the skyrmion to gradually shrink and eventually disappear^25,26^ (see Fig. 1(a,c)). The energy maximum along the MEP corresponds to a Bloch point-like texture, where the three central spins point opposite to each other (see snapshot ④ in Fig. 1).

The second mechanism corresponds to the skyrmion escaping through the boundary^29–31^ (see Fig. 1(b,c)). In the first section of the MEP, the skyrmion moves as a whole without changing its size and shape towards the boundary of the strip. This translational motion of the skyrmion involves almost no change in energy. Repulsive interaction^27,28^ with the twisted moments of the under-coordinated boundary sites causes the skyrmion to deform as it approaches the boundary and the energy to rise (see snapshot ⑤ in Fig. 1). At the energy maximum, the skyrmion touches the edge of the strip, locally untwisting the spins at the boundary (see snapshot ⑥ in Fig. 1). The skyrmion then forms an excitation at the edge of the strip, which resembles the tail of a spin spiral. It then leaves the sample by shrinking, which results in a linear decrease in energy along the MEP (see snapshots ⑦ and ⑧ in Fig. 1). Our calculations show that both mechanisms of skyrmion annihilation are realized in the system for the whole field range above *B*_F_, where skyrmions exist as metastable states.

Figure 1 also shows the evolution of the absolute value of the topological charge *Q* along the MEPs. For the collapse mechanism, *Q* is unity until the saddle point is reached. At this point, it drops to zero. For the escape mechanism, the drop in the topological charge is not as abrupt and an indication of an inflection point is seen at *Q* ≈ 0.5 where a meron is formed^35^. Under certain conditions, such excitations may constitute a long-lived, metastable state bound to the edge of the system^36^.

### Energy barriers

**Figure 2:**
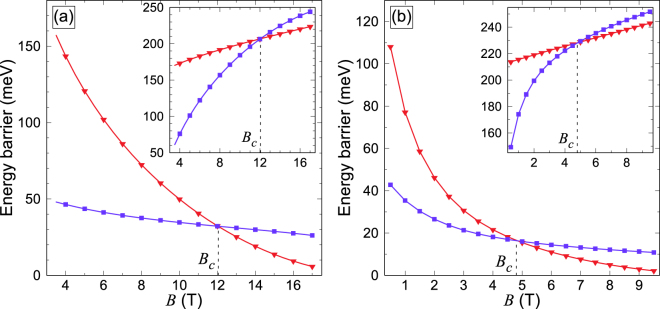
Energy barriers for skyrmion annihilation in a Pd/Fe/Ir(111) racetrack. Energy barrier for the skyrmion annihilation and nucleation (inset) in the interior (red curve, triangles) and at the boundary (purple curve, squares) of the Pd/Fe strip as a function of applied magnetic field strength, shown for the fcc (**a**) and hcp (**b**) stacking of the Pd layer. The curves intersect at the crossover field, *B*_*c*_.

From Fig. 1 it is clear that the energy barriers corresponding to the two pathways are not the same and they depend differently on the applied magnetic field. How the energy barriers vary with the external magnetic field is shown in Fig. 2 for the two annihilation mechanisms. At the external field at which single skyrmions emerge, *B* = *B*_F_, the energy required for the skyrmion to escape through the boundary is about one third of the energy required for it to collapse in the interior of the strip for both fcc and hcp stacking of the Pd layer. With increasing external field, barriers for both mechanisms decrease monotonically. However, the dependence is less pronounced for the escape mechanism. As a consequence, a crossover field, *B*_*c*_, exists, above which the lower energy barrier is provided by the collapse in the interior of the strip. Clearly, the magnitude of *B*_*c*_ depends on the value of the interaction parameters. For fcc-Pd/Fe/Ir(111), the crossover occurs at ${B}_{c}^{{\rm{fcc}}}=12$ T, while calculations for hcp-Pd/Fe/Ir(111) give 4.8 T for ${B}_{c}^{{\rm{hcp}}}$. Nevertheless, the presence of such a crossover between two mechanisms appears to be an inherent feature of skyrmions in confined geometries, and can be quite different in absolute values for different systems.

The determination of the energy barrier requires a very good knowledge of the entire energy landscape. In fact, we included DFT-derived exchange parameters for all atom pairs within 9 and 5 shells around each site for the fcc and hcp system, respectively (see Methods section for the detailed description of the first-principles model Hamiltonian). For comparison, we also performed calculations using the effective parameters deduced from the experimental data^34^ on field-dependent skyrmion profiles. In this case, the nearest-neighbor pairwise interactions were sufficient to model only a neighborhood of the local minimum of the energy landscape corresponding to the metastable skyrmion. Taking these effective parameters, then, the energy barriers become systematically smaller than those obtained with DFT-calculated interaction parameters, but the crossover still persists, with ${B}_{c}^{{\rm{eff}}}=2.6$ T (see Supplementary Fig. S2), which is significantly lower than both ${B}_{c}^{{\rm{fcc}}}$ and ${B}_{c}^{{\rm{hcp}}}$. Note however that effective parameters from ref.^34^ are not directly related to the shell-resolved parameters obtained from first principles calculations. It remains to be seen how long-range exchange interaction affects the crossover.

### Skyrmion lifetimes

In order to verify that the crossover deduced from the energy barriers truly represents a crossover between the annihilation mechanisms, the corresponding lifetimes and, therefore, the attempt frequency, *ν*, need to be evaluated. Within harmonic transition state theory^24^, the Arrhenius pre-exponential factor is calculated based on the quadratic approximation of the energy surface at the transition state saddle point and the skyrmion state minimum. The harmonic approximation breaks down for possible Goldstone modes, along which the energy of the system does not change, and a special treatment of such modes is needed^37–39^. In the case when the strip width *W* is much larger than the skyrmion size, there are two Goldstone modes at the skyrmion state minimum corresponding to a translational motion in the plane of the film. The volume associated with this motion is proportional to the size of the track (see Supplementary Note and Supplementary Table S1). At the saddle point corresponding to skyrmion collapse, changes in the spin structure are significant on the scale of the lattice constant. This removes the degeneracy of the in-plane translations. As a consequence, translational modes at the saddle point corresponding to skyrmion collapse cannot be treated as Goldstone modes at low temperature considered here. The number of saddle points equals the number of interstitial sites, which also scales with the system size. As a result, the pre-exponential factor for the collapse mechanism does not depend on the size of the system. The presence of Goldstone modes, however, introduces temperature dependence in the pre-exponential factor. Each Goldstone mode at the initial state contributes a factor of $\sqrt{2\pi {k}_{B}T}$ to the pre-exponential factor, while each Goldstone mode at the transition state gives a factor of $1/\sqrt{2\pi {k}_{B}T}$ (see Methods section). The prefactor for the collapse mechanism therefore scales with temperature as *ν*_c*ol*_(*T*) ∝ *T*. The saddle point for the boundary escape has one Goldstone mode corresponding to the translational motion of the excitation along the strip edge. As a result, the pre-exponential factor for the escape mechanism is inversely proportional to the width of the strip and scales with the square root of temperature, ${\nu }_{{\rm{esc}}}(T)\propto \sqrt{T}/W$. The results of the pre-exponential factor calculations for the 23.5 nm wide strip, several field strengths above the critical field for the low-temperature regime of 10 K are summarized in Table 1. Interestingly, the pre-exponential factor for the escape mechanism is almost insensitive to the field, but it changes strongly, by two orders of magnitude for the collapse mechanism within the same magnetic field range. Apparently, the changes in the entropy of the skyrmion state as the field strength is changed are compensated by analogous entropy changes in the transition state for the escape mechanism, while this compensation does not occur for the collapse mechanism. These results have important implications. If the skyrmion stability is mostly defined by the escape annihilation mechanism, a constant attempt frequency approach can be employed and an evaluation of the energy barrier is sufficient for estimating the lifetime. For the collapse mechanism, however, both the attempt frequency and the barrier must be calculated for accurate evaluation of the skyrmion lifetime. Dramatic changes in the Arrhenius pre-exponential factor have recently been observed experimentally for skyrmions in Fe_1−*x*_Co_*x*_Si system^40^.

**Table 1:** Pre-exponential factors for skyrmion annihilation in a Pd/Fe/Ir(111) racetrack.

fcc-Pd/Fe/Ir(111)			hcp-Pd/Fe/Ir(111)		
*B* (T)	*ν*_c*ol*_ (s^−1^)	*ν*_*esc*_ (s^−1^)	*B* (T)	*ν*_c*ol*_ (s^−1^)	*ν*_*esc*_ (s^−1^)
4	4.0 ⋅ 10^14^	1.2 ⋅ 10^10^	1	2.7 ⋅ 10^10^	1.1 ⋅ 10^9^
5	2.7 ⋅ 10^14^	1.1 ⋅ 10^10^	2	4.4 ⋅ 10^11^	1.8 ⋅ 10^9^
6	1.8 ⋅ 10^14^	1.1 ⋅ 10^10^	3	1.2 ⋅ 10^12^	2.4 ⋅ 10^9^
7	1.2 ⋅ 10^14^	1.1 ⋅ 10^10^	4	2.0 ⋅ 10^12^	2.9 ⋅ 10^9^
8	8.4 ⋅ 10^13^	1.1 ⋅ 10^10^	5	2.5 ⋅ 10^12^	3.3 ⋅ 10^9^
9	5.9 ⋅ 10^13^	1.0 ⋅ 10^10^	6	2.8 ⋅ 10^12^	3.7 ⋅ 10^9^
10	4.3 ⋅ 10^13^	1.0 ⋅ 10^10^	7	2.9 ⋅ 10^12^	4.0 ⋅ 10^9^
11	3.1 ⋅ 10^13^	1.0 ⋅ 10^10^	8	3.0 ⋅ 10^12^	4.3 ⋅ 10^9^
12	2.3 ⋅ 10^13^	1.0 ⋅ 10^10^	9	3.0 ⋅ 10^12^	4.7 ⋅ 10^9^
13	1.7 ⋅ 10^13^	1.0 ⋅ 10^10^	—	—	—
14	1.3 ⋅ 10^13^	1.0 ⋅ 10^10^	—	—	—
15	9.7 ⋅ 10^12^	1.1 ⋅ 10^10^	—	—	—
16	7.3 ⋅ 10^12^	1.2 ⋅ 10^10^	—	—	—
17	5.5 ⋅ 10^12^	1.5 ⋅ 10^10^	—	—	—

The pre-exponential factors for the skyrmion collapse, *ν*_c*ol*_, and escape, *ν*_e*sc*_, in a 23.5 nm wide Pd/Fe strip on Ir(111) for several applied magnetic field strengths and a temperature of 10 K. Calculations have been carried out for both fcc and hcp stackings of the Pd layer.

The relaxation time associated with each annihilation mechanism, *τ*_col_ and *τ*_esc_, can now be calculated using Eq. (1). In addition to a strong, exponential dependence on temperature, both *τ*_col_ and *τ*_esc_ are characterized by a weak power dependence on temperature originating from the pre-exponential factor. Moreover, *τ*_esc_ scales with the strip width. This explicitly demonstrates that boundaries are less important for the stability of skyrmions in wider strips, as expected. In Fig. 2 the calculated results of the dependence of the skyrmion lifetime on the applied magnetic field and temperature are presented for a 23.5 nm wide strip, which is roughly five times larger than the skyrmion size at the critical field, *B*_F_. Both annihilation mechanisms contribute to the skyrmion stability, and the lifetime, *τ*, is related to *τ*_col_ and *τ*_esc_ according to

2 $$1/\tau =1/{\tau }_{{\rm{col}}}+1/{\tau }_{{\rm{esc}}},$$

which follows from a general theory of two uncorrelated processes. Overall, for a given external field the contour graph exhibits an exponential dependence of the skyrmion lifetime on the temperature, and the lifetime changes at the critical field, *B*_F_, from the age of the universe to microseconds in a narrow temperature range of 25 K, which is about 10% of the critical temperature for the Pd/Fe/Ir system^18^. Two regions can be distinguished, corresponding to the two mechanisms of skyrmion annihilation. In the ‘collapse’ region, *τ*_col_ < *τ*_esc_, the skyrmion annihilation is dominated by the collapse mechanism, while in the ‘escape’ region the lifetime is mostly defined by *τ*_esc_. The two regions are separated by the crossover line defined as *τ*_col_ = *τ*_esc_ = 2*τ*. Notice that the crossover line is not parallel to the temperature axis, indicating that the crossover field is temperature dependent. At low temperature, the crossover field coincides with that of the lowest energy barrier. At higher temperature, entropy comes into play, leading to a decrease in the crossover field. Although fcc and hcp stackings of the Pd layer result in different skyrmion lifetime, the results for both stackings share the same characteristic features (see also the results for the effective, nearest neighbor Hamiltonian from ref.^34^ in Supplementary Fig. S3). The decrease in the skyrmion lifetime with temperature and applied magnetic field is also consistent with the trends obtained from the telegraph noise measurements of Romming *et al*.^13^, although reported data might be insufficient for direct comparison of the lifetimes.

## Discussion

**Figure 3:**
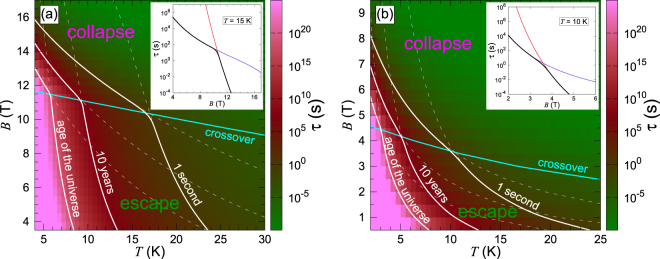
Lifetime of a skyrmion in a Pd/Fe/Ir(111) racetrack. Contour plot of the calculated lifetime of an isolated skyrmion in a 23.5 nm wide strip as a function of applied magnetic field strength and temperature, shown for the fcc (**a**) and hcp (**b**) stacking of the Pd layer. White contour lines have a characteristic cusp due to the crossover between collapse and escape mechanism indicated by the cyan line. Above the crossover line, the skyrmion lifetime is mostly defined by the collapse mechanism, but the escape mechanism dominates below the crossover line. White dashed lines indicate isochronal contours of the collapse and escape lifetimes. Insets show the cut of the contour plot at *T* = 15 K (**a**) and *T* = 10 K (**b**); in the insets, annihilation time due to collapse in the interior, escape through the boundary and total skyrmion lifetime are shown with red, purple and black curves, respectively.

Our results show that boundaries have a crucial impact on the skyrmion stability in racetrack strips at low magnetic fields. If the system boundaries are not included in the analysis, we find that the skyrmion lifetime in the Pd/Fe/Ir(111) system at *B* = *B*_F_, i.e. when metastable skyrmions emerge at a ferromagnetic background, is expected to be ten years at *T* ≈ 30–35 K for both fcc and hcp stacking of the Pd layer (see dashed contour lines of *τ*_c*ol*_ in Fig. 3). This, by the way, is significantly different from results deduced from effective Hamiltonian with nearest-neighbor pairwise interactions and Monte Carlo simulations mimicking overdamped spin dynamics in an extended Pd/Fe/Ir(111) film at elevated temperature^19^. By fitting the simulation parameters to experimental data and extrapolating to low temperature, the Monte Carlo simulations indicated mean lifetime on the order of years at around 19 K, whereas our calculations predict a lifetime comparable to the age of the universe at this temperature. When the width of the strip is taken into account, the temperature at which the mean lifetime of a skyrmion becomes acceptably long for information storage is lowered even further. For example, a lifetime of ten years is achieved at *B* = *B*_F_ only below 15 K in a strip that is five times wider than the size of the skyrmion, $W\simeq 5\,{L}_{{\rm{s}}}$. The lifetime for the escape process is proportional to the strip width, *τ*_esc_ ∝ *W*, which is a consequence of smaller probability for a skyrmion to reach the boundary in a wider strip. Thus, the above temperature can be raised to 30 K by increasing the strip width. A rough estimate gives however that this would require strips wider by a factor larger than 10^8^, which are totally unrealistic in practise. Clearly, for any practical purpose, the escape mechanism is the most important one for skyrmion annihilation and nucleation in the Pd/Fe/Ir systems. In order to enhance the skyrmion stability at magnetic fields at which metastable skyrmions emerge, decoration of some kind at the boundaries of the strip is needed. For example, Stosic *et al*. predict that the energy barrier for skyrmion annihilation at the boundary is enhanced by a local decrease in the DM interaction at the edge of the track^29^.

At larger applied fields, the collapse mechanism becomes increasingly important and completely dominates the skyrmion annihilation process above the crossover field. The crossover between the annihilation mechanisms is to a large extent due to the crossover of the lowest energy barrier (compare insets in Figs 2 and 3), which has a simple interpretation. Consider the effect of skyrmion size, *L*_*s*_. The energy barrier associated with the collapse mechanism is proportional to the number of spins that need to be flipped in this process and, therefore, scales with ${L}_{{\rm{s}}}^{2}$. As expected, the barrier goes to zero as the skyrmion size approaches the size of the saddle point excitation, which essentially represents a Bloch point-like defect. The size of the defect is small but finite and it appears to be roughly field independent. On the other hand, the energy barrier for the escape mechanism is defined by the number of spins that need to be untwisted at the boundary, not by the shrinking of the skyrmion. When the skyrmion leaves the system, it untwists the boundary locally, with the number of spins involved scaling as ~*L*_s_. Since the skyrmion size is a monotonous function of the applied magnetic field^1,2,34^, the weaker dependence of the escape barrier on *L*_s_ results in a weaker dependence on *B*. In contrast to the collapse mechanism, the energy barrier for the escape mechanism remains finite as the magnetic field approaches an upper critical field *B*_*u*_, at which the skyrmion solution becomes unstable, due to the finite size of the skyrmion and the boundary twist which is present as the field reaches the value *B*_*u*_^28,41^. This explains why curves Δ*E*_col_(*B*) and Δ*E*_esc_(*B*) must intersect. This conclusion is rather general and should be valid for any finite-size skyrmion system.

It is interesting to compare the barrier for the skyrmion creation and annihilation with the well-known energy of a vanishing Belavin-Polyakov (BP) vortex with topological charge |*Q*| = 1^42,43^. Such a magnetic state is reminiscent of a skyrmion of vanishing size–a spin texture through which the skyrmion can collapse in our model. The corresponding energy serves as a standard scale for energy barriers. With the exchange energy of a two-dimensional system described as

$$E(\overrightarrow{m})=tA{\int }_{{{\mathbb{R}}}^{2}}[|\overrightarrow{{\rm{\nabla }}}{m}_{x}{|}^{2}+|\overrightarrow{{\rm{\nabla }}}{m}_{y}{|}^{2}+|\overrightarrow{{\rm{\nabla }}}{m}_{z}{|}^{2}]d\overrightarrow{r},$$

where *A* is the exchange stiffness and *t* is the magnetic layer thickness, the energy of the BP vortex with respect to the ferromagnetic state amounts to *E*_BP_ = 8*πtA*^42,43^. Taking an experimentally deduced value for *A* = 2.0 pJ/m^34^, and DFT-calculated value for *t* = 4.08 Å^14^, we obtain a nucleation barrier of 128 meV, which is about 60% larger than the barrier height calculated with the atomistic spin Hamiltonian and the equivalent effective, nearest-neighbor exchange interaction parameter, *J*_eff_ = 2*At*/(3)^1/2^ ^44^ (see the inset in Supplementary Fig. S2). The agreement between the models is surprisingly good, given that the micromagnetic model completely ignores the structure of the transition state on the atomic lattice scale. We conclude that the continuum-model estimate can be used to predict the characteristic energy scale of the barrier, but is insufficient for the quantitative determination of the skyrmion stability and lifetime.

The present study dealt with infinite racetracks, but if the length of the track is finite, the skyrmions would also be able to escape through the track ends, resulting in an additional contribution to the escape rate that scales inversely with the track length (analogous to the contribution from the escape through the sides of the track, which scales inversely with the track width). Note that this extra contribution to the skyrmion annihilation rate would not affect other relevant annihilation mechanisms, including collapse in the interior of the track and escape through the track sides.

In this article we focused on the long-lifetime limit as relevant for technology associated with the low-temperature regime for the Pd/Fe/Ir(111) systems. Here, we briefly comment on the short lifetime regimes. Typical magnetic time scales are microsecond (MHz), nanosecond (GHz) and picosecond (THz) regimes, related to skyrmion core gyration dynamics, magnon excitation and spin precession, respectively. Staying at external magnetic fields $B\gtrsim {B}_{{\rm{F}}}$, where single skyrmions are metastable with respect to a ferromagnetic background, and large sample sizes, where the escape mechanism becomes irrelevant, the lifetime of the single skyrmion matches these timescales at around 80 K, 140 K, and 200 K, respectively. Thus, in case of interest in the fabrication of frequency-tunable spin-torque vortex or skyrmion oscillators^45,46^, the operation temperature should be significantly below 80 K. We note that such devices–operating at several tesla and such low temperature–would hardly be practical. When skyrmion annihilation and creation interferes with magnon excitations, also the excitation of short-lived antiskyrmions or skyrmions of different charges is possible. This is consistent with the intermediate phase regime of skyrmions and antiskyrmions introduced in ref.^18^. The spin-precession time coincides with the lifetime of skyrmions around the critical temperature of the ferromagnetic phase to the paramagnetic phase. In that situation, of course the lifetime is not anymore a relevant concept.

The Pd/Fe/Ir(111) system where skyrmions are stable only at low temperature and high magnetic field is probably not directly relevant for technological applications. But, it is one of the best studied skyrmionic systems, and is chosen here to illustrate consistent calculations of skyrmion lifetime from first principles, without employing phenomenological parameters. We believe that the theoretical approach used here, which combines a statistical description of skyrmion stability with an atomistic spin Hamiltonian parametrized from first-principles electronic structure calculations gives valuable insight into the main mechanisms governing the lifetime of skyrmions and provides a tool for predictive materials design for skyrmion-based information technology. The exponential temperature dependence, the importance of the finite width on the lifetime in small field and the crossover effect at higher field combined with a finite temperature range for skyrmion lifetimes that are long enough for technological applications are likely general features of skyrmions in finite systems, including multilayer heterostructures, where skyrmions exist at room temperature^7,47,48^.

## Methods

### Simulated system

We model the Pd/Fe film as a single monolayer of classical spins on a hexagonal lattice defined by the Ir(111) substrate. The lattice constant is 2.7 Å^14^. While the Fe layer follows the fcc stacking of the Ir(111) surface, both fcc and hcp stackings for the Pd atoms have been observed experimentally^12^ and DFT calculations have been carried out for both types of layers, resulting in two sets of magnetic interaction parameters^17^. The two structures are referred to as fcc-Pd/Fe/Ir(111) and hcp-Pd/Fe/Ir(111), respectively.

A magnetic racetrack is simulated by applying periodic boundary conditions along one of the lattice-bond directions, while imposing open boundary conditions along the orthogonal direction. The size of the computational domain was chosen to be large enough for the isolated skyrmion at the center of the strip not to be affected by the boundaries. The width of the strip is taken to be *W* = 23.5 nm, which is roughly 5 times larger than the skyrmion size at the critical field *B*_F_.

### First-principles model Hamiltonian

The energy landscape of the Pd/Fe monolayer strip on Ir(111) is described by the following atomistic Hamiltonian:

3 $$\begin{array}{rcl}E & = & -\sum _{i,j}{J}_{ij}^{X}{\overrightarrow{m}}_{i}\cdot {\overrightarrow{m}}_{j}-\sum _{i,j}{\overrightarrow{D}}_{ij}^{X}\cdot [{\overrightarrow{m}}_{i}\times {\overrightarrow{m}}_{j}]\\  &  & -{K}^{X}\sum _{i}{({\overrightarrow{m}}_{i}\cdot {\hat{e}}_{K})}^{2}-{\mu }_{s}\overrightarrow{B}\sum _{i}{\overrightarrow{m}}_{i}.\end{array}$$

Here, summation in the first two terms runs over distinct pairs of atoms, ${\overrightarrow{m}}_{i}$ is a unit vector defining the orientation of the magnetic moment at site *i*. The superscript *X* indicates the parameter set: *X* = fcc for the fcc-Pd/Fe/Ir(111) system, while *X* = hcp for the hcp-Pd/Fe/Ir(111) one. The DM vector, ${\overrightarrow{D}}_{ij}$, is a three-dimensional vector with components in the monolayer plane that point perpendicular to the bond connecting sites *i* and *j* and small positive and negative components normal to the surface. Since these normal components average out after summation over all pairs, they are neglected here. The unit vector ${\hat{e}}_{K}$ defining the easy axis as well as the external magnetic field $\overrightarrow{B}$, point perpendicular to the film plane. Shell-resolved exchange interaction parameters, *J*_*ij*_, DM interaction parameters, *D*_*ij*_, anisotropy constant, *K* as well as on-site magnetic moment, *μ*_*s*_, obtained from first-principles calculations were taken from ref.^17^. In order to describe the energy of the states along the MEP we included the interaction parameters due to exchange, *J*_*ij*_, for the pairwise contributions including up to 9, 5 shells with in total 72, 36 pairs per atom for the fcc, hcp stacked Pd/Fe/Ir system, respectively. We do not explicitly include dipole-dipole interactions. However, for ultrathin films this contribution to the energy can be effectively included into the magnetocrystalline anisotropy energy^3,26,49^.

### Evaluation of annihilation rates

The rate of skyrmion annihilation, which defines the lifetime, is calculated using harmonic transition state theory for magnetic systems^24^ extended to include the presence of Goldstone modes. One of the basic assumptions of transition state theory is that Boltzmann distribution is established in the region of configuration space that corresponds to the initial state before the system escapes due to thermal fluctuations. This assumption is justified when the escape events are rare on the time scale of magnetization dynamics of the system. The theory predicts an Arrhenius dependence of the rate on temperature (see Equation (1)), where the activation energy is given by the energy difference between the relevant saddle point and a local minimum on the energy surface corresponding to the initial state, while the pre-exponential factor is defined by the curvature of the energy surface at the saddle point and at the initial state minimum. If no Goldstone modes are present in the system, the Boltzmann average entering the equation for the transition rate is computed using Gaussian integration of the distribution function, *ρ* = *C* exp[−*E*/(*k*_B_*T*)], over all relevant degrees of freedom, resulting in the factor ∝ (2*πk*_B_*T*)^−*N*^ for a system of *N* spins. The same factor enters the normalization constant, *C* ∝ (2*πk*_B_*T*)^*N*^. As a result, the temperature dependence cancels out in the expression for the attempt frequency^24^. Gaussian integration breaks down for possible Goldstone modes, along which energy of the system is (nearly) constant. Therefore, such modes must be treated separately. Specifically, integration of the distribution function over a Goldstone mode gives the volume in the phase space associated with the mode. If the numbers of Goldstone modes are not the same at the minimum and at the saddle point, some of the factors $\sqrt{2\pi {k}_{{\rm{B}}}T}$ resulting from the Gaussian integration remain uncompensated and the pre-exponential factor acquires a power dependence on temperature. Specifically, the pre-exponential factor is given by the following equation, generalized to include Goldstone modes:

4 $$\nu =\frac{\gamma }{2\pi }\frac{{V}_{{\rm{S}}{\rm{P}}}}{{V}_{min}}{(2\pi {k}_{{\rm{B}}}T)}^{({P}_{min}-{P}_{{\rm{S}}{\rm{P}}})/2}\sqrt{\sum _{i}^{\prime} \frac{{a}_{i}^{2}}{{\varepsilon }_{i}}}\sqrt{\frac{{\rm{d}}{\rm{e}}{\rm{t}}\,{{\bf{H}}}_{min}}{{\rm{d}}{\rm{e}}{\rm{t}}^{\prime} {{\bf{H}}}_{{\rm{S}}{\rm{P}}}}}.$$

Here, *γ* is the gyromagnetic ratio, det **H**_*κ*_, *P*_*κ*_ and *V*_*κ*_ denote the product of the eigenvalues of the Hessian matrices **H**_*κ*_, numbers of Goldstone modes and volumes associated with the Goldstone modes, respectively, at the stable state minimum (*κ* = min) and at the saddle point (*κ* = SP), index *i* = 1, …, 2*N* labels degrees of freedom in the system, *ε*_*i*_ are the eigenvalues of the Hessian at the saddle point, and *a*_*i*_ are expansion coefficients in the linearized equation for the unstable mode derived from the Landau-Lifshitz equations of motion for 2*N* degrees of freedom of the system^24^. The eigenvalues of the Hessian matrices have been computed using the Intel Math Kernel Library^50^. The terms corresponding to the Goldstone modes are omitted in the determinants and the summation. The terms associated with the unstable mode are omitted as well, as indicated by the prime superscript.

### Minimum energy path calculations

Calculation of minimum energy paths (MEPs) is needed for a definitive identification of transition state saddle points, which define the transition rates within the harmonic transition state theory. An MEP between two minima is the path in configuration space which lies lowermost on the energy surface. Following an MEP means rotating spins in an optimal way, so that the energy is minimal with respect to all degrees of freedom perpendicular to the path. The MEP not only gives the location of the saddle point, which corresponds to a maximum along the MEP, but also provides detailed information about the transition mechanism, important quantitative knowledge, which is not easily accessible in the spin dynamics simulations. A Geodesic Nudged Elastic Band (GNEB) method^25,51^ is used to find MEPs of skyrmion annihilation. The GNEB method involves taking some initial guesses of the path represented by a discrete chain of states of the system, and systematically bringing that to the nearest MEP by zeroing the transverse component of the gradient force at each point along the path, as described in the following. In order to distribute the states evenly along the path, virtual springs are introduced between adjacent states. At each state, a local tangent to the path is estimated and the GNEB force guiding the states towards the MEP is defined as the sum of the transverse component of the negative energy gradient and the component of the spring force along the tangent. The positions of states are then adjusted using some optimization algorithm so as to zero the GNEB force. In the method, both the GNEB force and the path tangent are defined in the local tangent space to the curved configuration space, which is needed to satisfy constraints on the length of magnetic moments and to properly decouple the perpendicular component of the energy gradient from the spring force^25^.

### Data availability

All data generated or analysed during this study are included in the article and its Supplementary Information file.

## Electronic supplementary material

Supplementary information
