# Supplementary material for: Lifetime of racetrack skyrmions
Source: Sci Rep. 2018 Feb 21;8:3433. doi: 10.1038/s41598-018-21623-3 (PMC5821818; doi:10.1038/s41598-018-21623-3)
Supplement: Supplementary file 1 — Supplementary information [file 41598_2018_21623_MOESM1_ESM.pdf]

Supplementary information for "Lifetime of racetrack skyrmions"

Pavel F. Bessarab,[1,2,*] Gideon P. Müller,[1,3] Igor S. Lobanov,[2] Filipp N. Rybakov,[4] Nikolai S. Kiselev,[3] Hannes Jónsson,[1,5] Valery M. Uzdin,[6,2] Stefan Blügel,[3] Lars Bergqvist,[7,8] and Anna Delin[7,8,9,†]

[1] *Science Institute of the University of Iceland, 107 Reykjavik, Iceland*

[2] *ITMO University, 197101 St. Petersburg, Russia*

[3] *Peter Grünberg Institut and Institute for Advanced Simulation, Forschungszentrum Jülich and JARA, D-52425 Jülich, Germany*

[4] *KTH Royal Institute of Technology, Stockholm SE-10691, Sweden*

[5] *Aalto University, FI-00076 Espoo, Finland*

[6] *Department of Physics, St. Petersburg State University, St. Petersburg, 198504, Russia*

[7] *Department of Applied Physics, School of Engineering Sciences, KTH Royal Institute of Technology, Electrum 229, SE-16440 Kista, Sweden*

[8] *SeRC (Swedish e-Science Research Center), KTH Royal Institute of Technology, SE-10044 Stockholm, Sweden*

[9] *Department of Physics and Astronomy, Uppsala University, Box 516, SE-75120 Uppsala, Sweden*

(Dated: January 28, 2018)

* bessarab@hi.is

† annadel@kth.se

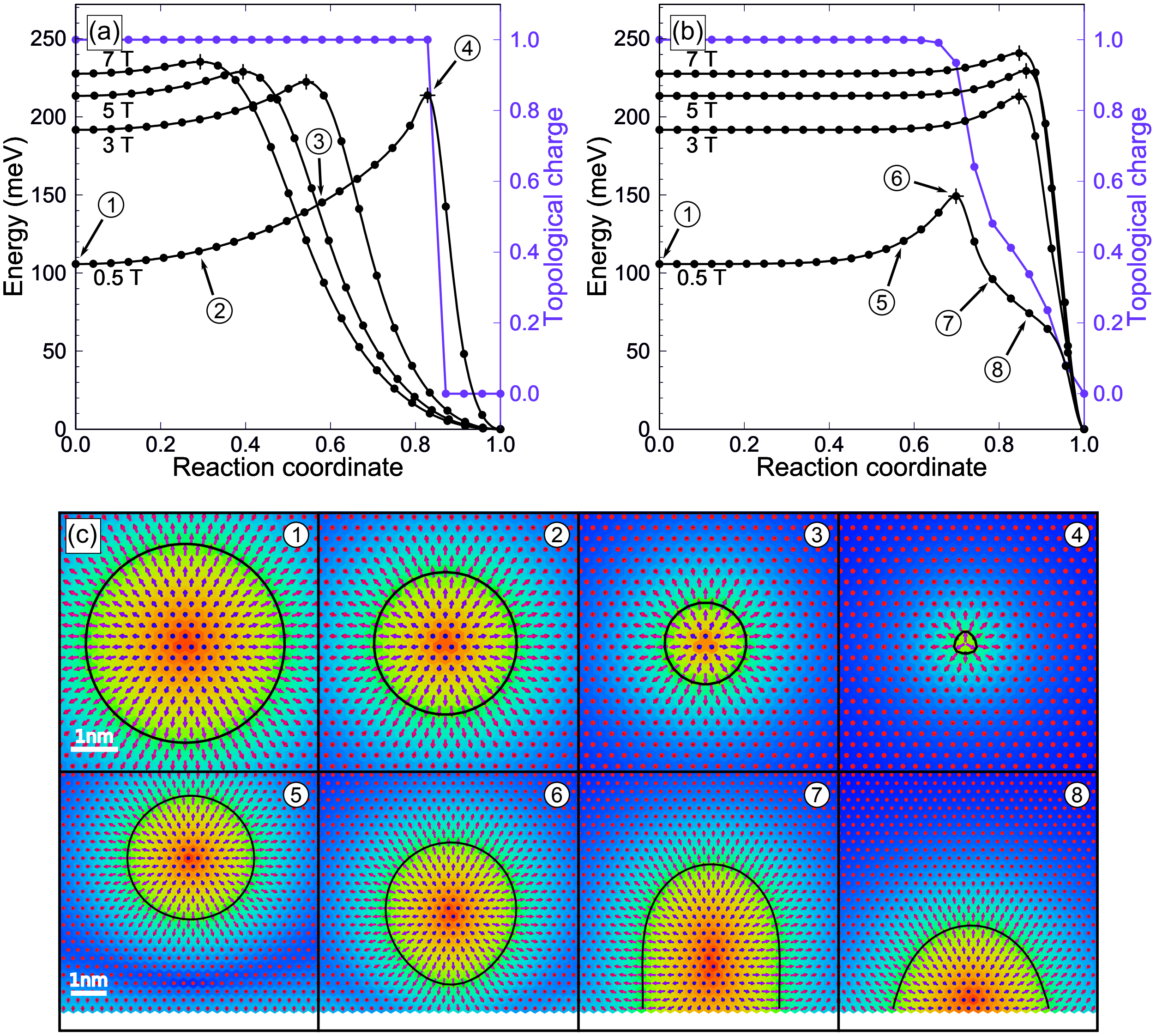

Supplementary Figure S1. Mechanisms of the skyrmion annihilation in a hcp-Pd/Fe/Ir(111) racetrack. Energy variation along the minimum energy paths (MEPs) for radial collapse of the skyrmion at the interior of the strip (a) and escape of the skyrmion through the boundary (b), shown for four values of applied magnetic field. The filled circles show position of the intermediate states along the annihilation paths, while crosses indicate energy maxima along the MEPs. Variation of the topological charge along the MEPs is shown with a purple line for $B = 0.5$ T. The reaction coordinate is defined as the normalized displacement along the MEP. The starting- and end-points of the reaction coordinate are the skyrmion and ferromagnetic states, respectively. The encircled numbers label the images for which spin configurations are shown in the lower panel (c). The background color indicates the value of the out-of-plane component of the magnetic vectors (red $\leftrightarrow$ up, blue $\leftrightarrow$ down). Black solid lines show the contour where the out-of-plane component of magnetization vanishes.

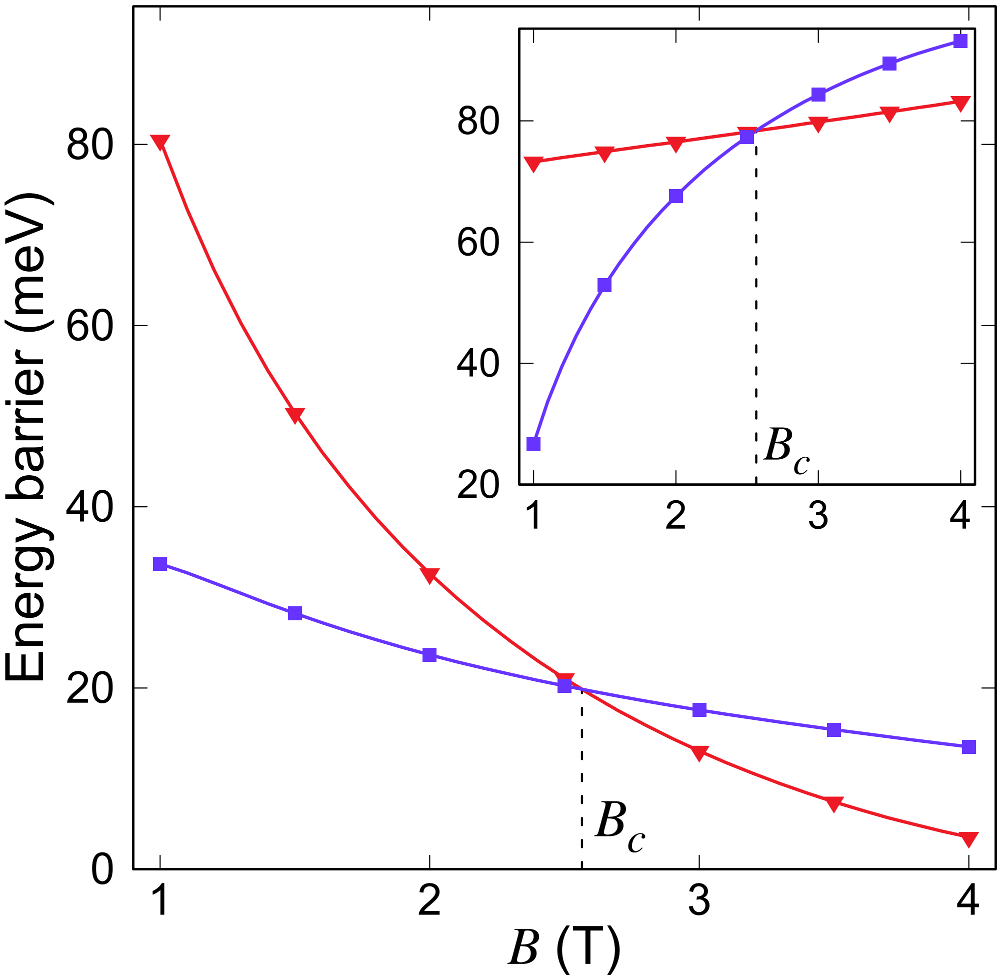

Supplementary Figure S2. Energy barriers for skyrmion annihilation in a Pd/Fe/Ir(111) racetrack described by the effective, nearest neighbor exchange model. Energy barrier for the skyrmion annihilation and nucleation (inset) at the interior (red curve, triangles) and at the boundary (purple curve, squares) of the Pd/Fe strip as a function of applied field strength. The curves intersect at the crossover field, B_c. Parameters of the effective Hamiltonian are taken from Ref. [34] of the main text.

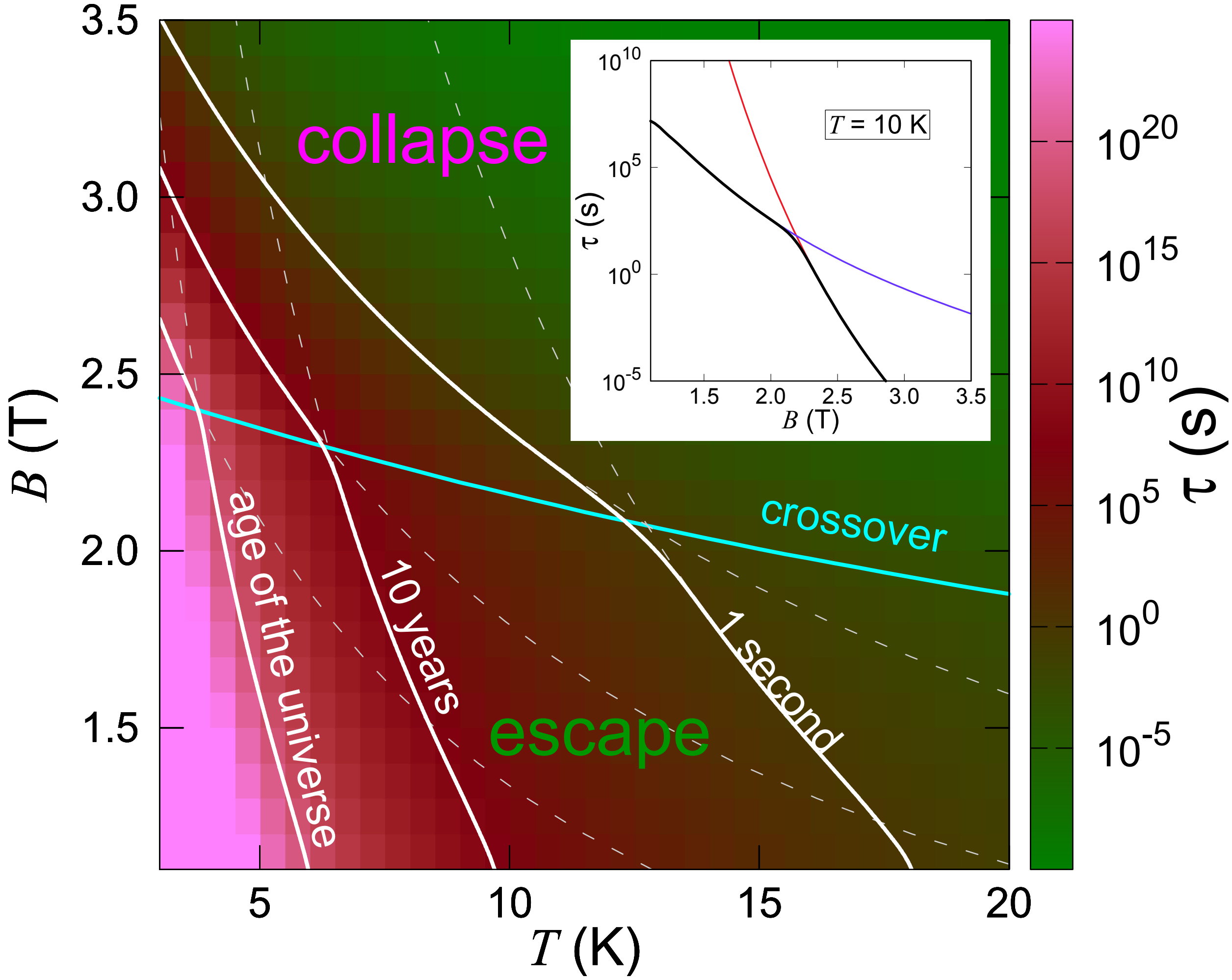

Supplementary Figure S3. Lifetime of a skyrmion in a Pd/Fe/Ir(111) racetrack described by the effective, nearest neighbor exchange model. Contour plot of the calculated lifetime of an isolated skyrmion in a 23.5 nm wide strip as a function of applied magnetic field strength and temperature. White contour lines have a characteristic cusp due to the crossover indicated by the cyan line. Above the crossover line, the skyrmion lifetime is mostly defined by the collapse mechanism, but the escape mechanism dominates below the crossover line. White dashed lines indicate contours of the collapse and escape relaxation times. Inset shows the cut of the contour plot at $T = 10$ K; in the inset, annihilation time due to collapse at the interior, escape through the boundary and total skyrmion lifetime are shown with red, purple and black curves, respectively. Parameters of the effective Hamiltonian are taken from Ref. [34] of the main text.

Supplemenary Note. Contribution of the Goldstone modes

Integration of the distribution function over a Goldstone mode is non-Gaussian and results in the volume of the mode. For a skyrmion in the magnetic strip, the Goldstone modes correspond to pure translations of the magnetic configuration. Consequently, the volumes of the modes can be obtained by the integration over spatial coordinate [S1], as described below. A localized magnetic texture such as the skyrmion state or the saddle point state for the skyrmion annihilation can be described by a set of unit vectors, $\boldsymbol{m} = (\vec{m}_1, \vec{m}_2, \dots, \vec{m}_N)$, where N is a number of spins in the system. Let $\vec{r}$ be the position of the localized state, e.g. position of the skyrmion center. Translation of the magnetic texture along direction $\vec{e}_x$ by a distance dx can be described by:

$$\boldsymbol{m}(\vec{r} + \vec{e}_x dx) - \boldsymbol{m}(\vec{r}) = \frac{d\boldsymbol{m}(\vec{r})}{dx} dx. \tag{S1}$$

Same changes in the magnetic structure are generated by the displacement along the Goldstone mode $\boldsymbol{Q}(\vec{r})$. Therefore, one can equally well write:

$$\boldsymbol{m}(\vec{r} + \vec{e}_x dx) - \boldsymbol{m}(\vec{r}) = \boldsymbol{Q}(\vec{r}) dq. \tag{S2}$$

The Goldstone mode is proportional to $\frac{d\boldsymbol{m}(\vec{r})}{dx}$:

$$A\boldsymbol{Q}(\vec{r}) = \frac{d\boldsymbol{m}(\vec{r})}{dx}, \tag{S3}$$

where factor A is fixed by the normalization condition, $|\boldsymbol{Q}(\vec{r})| = 1$:

$$A = \left|\frac{d\boldsymbol{m}(\vec{r})}{dx}\right| = \left(\left|\frac{d\vec{m}_1(\vec{r})}{dx}\right|^2 + \left|\frac{d\vec{m}_2(\vec{r})}{dx}\right|^2 + \ldots + \left|\frac{d\vec{m}_N(\vec{r})}{dx}\right|^2\right)^{1/2}. \tag{S4}$$

Supplementary Equations (S1)-(S4) make it possible to replace integration over the Goldstone mode by an integration over spatial coordinate in order to find the volume of the mode V_q:

$$V_q = \int dq = A \int_0^{L_x} dx = A L_x, \tag{S5}$$

where L_x is the system size along $\vec{e}_x$. According to Supplementary Equation (S5), normalization factor A can be interpreted as the Goldstone mode volume per unit length.

For a magnetic configuration defined on a lattice, normalization factor A can be obtained by the evaluation of the finite-difference representation of derivatives in the RHS of Supplementary Equation (S4). Specifically, if $\vec{e}_x$ is chosen to be along the lattice vector $\vec{a}$:

$$A \approx \frac{1}{a} \left| \boldsymbol{m}(\vec{r} + \vec{a}) - \boldsymbol{m}(\vec{r}) \right| = \frac{1}{a} \left(\sum_{i=1}^{N} \left| \vec{m}_i(\vec{r} + \vec{a}) - \vec{m}_i(\vec{r}) \right|^2 \right)^{1/2}, \tag{S6}$$

where a is a lattice constant. Values of the Goldstone mode volume per unit length for the skyrmion state minimum and saddle point state corresponding to the boundary escape and several magnetic field strengths above the critical field are summarized in Supplementary Table S1.

Supplementary Table S1. Goldstone mode volumes per unit length for skyrmions in a Pd/Fe/Ir(111) racetrack. The Goldstone mode volumes for the skyrmion state minimum, A_{min}, and saddle point state corresponding to the boundary escape, A_{SP}, in a Pd/Fe strip on Ir(111) for several applied magnetic field strengths. Results are given for both fcc and hcp stackings of the Pd layer.

fcc-Pd/Fe/Ir(111)			hcp-Pd/Fe/Ir(111)		
B (T)	A_{min} (1/a)	A_{SP} (1/a)	B (T)	A_{min} (1/a)	A_{SP} (1/a)
4	4.38	4.79	1	4.39	4.49
5	4.31	4.64	2	4.14	4.21
6	4.26	4.54	3	4.03	4.09
7	4.22	4.47	4	3.98	4.04
8	4.18	4.41	5	3.95	4.01
9	4.15	4.36	6	3.93	3.98
10	4.13	4.32	7	3.92	3.96
11	4.10	4.28	8	3.90	3.95
12	4.08	4.24	9	3.89	3.93
13	4.05	4.20	-	-	-
14	4.02	4.16	-	-	-
15	4.00	4.12	-	-	-
16	3.97	4.07	-	-	-
17	3.94	4.01	-	-	-

[S1] H.B. Braun, Phys. Rev. B **50**, 16501 (1994).
